# Longitudinal Outcomes and Ribavirin Use in Lung Transplant Recipients with Respiratory Syncytial Virus or Human Metapneumovirus Infection: A Real-World Multicenter Cohort Study

**DOI:** 10.3390/life16081323

**Published:** 2026-08-12

**Authors:** Miguel Jiménez-Gómez, Beatriz Montull-Veiga, Víctor Manuel Mora-Cuesta, Eva Revilla-López, Myriam Aguilar-Pérez, Alicia de-Pablo-Gafas, Juan Margallo-Iribarnegaray, Carlos Andrés Quezada-Loaiza, Ana Hernández-Voth, Francisco López-Medrano, María Ruiz-Rodríguez, Rodrigo Alonso-Moralejo

**Affiliations:** 1 Lung Transplant Unit, Pulmonology Department, 12 de Octubre University Hospital, 28041 Madrid, Spain; migueljimenezgomez@gmail.com (M.J.-G.); juanmargalloi@gmail.com (J.M.-I.); andresquezadal@gmail.com (C.A.Q.-L.); mruizrodriguez3@salud.madrid.org (M.R.-R.); ralonsomoralejo@gmail.com (R.A.-M.); 2 Lung Transplant Unit, Pulmonology Department, La Fe de Valencia University Hospital, 46026 Valencia, Spain; beatrizmontullveiga@gmail.com; 3 Lung Transplant Unit, Pulmonology Department, Marqués de Valdecilla University Hospital, 39008 Santander, Spain; vmoracuesta@gmail.com; 4 Lung Transplant Unit, Pulmonology Department, Vall d’Hebron University Hospital, 08035 Barcelona, Spain; eva.revilla@vallhebron.cat; 5 Lung Transplant Unit, Pulmonology Department, Puerta de Hierro University Hospital, 28222 Madrid, Spain; myriamaguipe@yahoo.es; 6 Instituto de Investigación Hospital “12 de Octubre” (i+12), 12 de Octubre University Hospital, 28041 Madrid, Spain; anahvoth@gmail.com (A.H.-V.); flmedrano@yahoo.es (F.L.-M.); 7 Mechanical Ventilation Unit, Pulmonology Department, 12 de Octubre University Hospital, 28041 Madrid, Spain; 8 Centro de Investigación Biomédica en Red de Enfermedades Infecciosas (CIBERINFEC), Instituto de Salud Carlos III, 28029 Madrid, Spain; 9 Unit of Infectious Diseases, 12 de Octubre University Hospital, School of Medicine, Universidad Complutense, 28041 Madrid, Spain

**Keywords:** ribavirin, respiratory viral infection, lung allograft dysfunction, respiratory syncytial virus, human metapneumovirus

## Abstract

Respiratory syncytial virus (RSV) and human metapneumovirus (hMPV) are clinically relevant pathogens in lung transplant (LT) recipients, but their impact on lung function and the benefit of ribavirin remains uncertain. We conducted a prospective multicenter observational cohort study of adult LT recipients with RSV or hMPV infection diagnosed between 2021 and 2024 at five centers, with follow-up for up to 12 months. Ribavirin was prescribed at the treating physician’s discretion. Multivariable analysis assessed the association between ribavirin and percentage change in forced expiratory volume in the first second (FEV_1_) at 90 days, using FEV_1_ three months before infection as baseline and adjusting for baseline FEV_1_, pre-existing chronic lung allograft dysfunction, and time since transplantation. Seventy-six patients were included; 60 (78.9%) had RSV and 16 (21.1%) hMPV. Lower respiratory tract infection occurred in 48.7%, and an acute ≥10% FEV_1_ decline at 14 days was observed in 29.3%. Among patients with lower respiratory tract infection, 45.9% received corticosteroids alone and 37.8% corticosteroids plus ribavirin. No statistically significant between-group differences in allograft dysfunction or mortality were observed. RSV and hMPV infections after LT were frequently associated with lower respiratory involvement and lung function decline. In this observational cohort, ribavirin use was not independently associated with 90-day FEV_1_ change; however, residual confounding and limited statistical power preclude conclusions regarding treatment efficacy.

## 1. Introduction

Respiratory viral infections (RVIs) represent a major source of morbidity in solid organ transplant recipients. Over a median follow-up of 3.4 years, the cumulative incidence of RVI reached approximately 60% in lung transplant (LT) recipients, compared with 12% in recipients of other transplant solid organs [1]. This increased susceptibility reflects the combination of lifelong immunosuppression, direct exposure of the allograft to the external environment, and structural and functional alterations following transplantation [2].

Clinical presentation in transplant recipients may be atypical, frequently with mild symptoms and the absence of fever, which may delay diagnosis. Molecular nucleic acid amplification assays currently represent the most sensitive diagnostic tools and allow the simultaneous detection of a broad range of respiratory pathogens [3]. Beyond the acute infectious episode, RVIs are associated with important indirect consequences, including secondary bacterial or fungal infections [3] and potential immunologic complications affecting the lung allograft [4].

Among RVI, respiratory syncytial virus (RSV) and human metapneumovirus (hMPV) are increasingly recognized as clinically relevant pathogens in LT recipients [5]. Both are enveloped ribonucleic acid (RNA) viruses belonging to the Pneumoviridae family and share similar epidemiological and clinical characteristics. The reported incidence of these viruses in adult LT patients ranges from approximately 4.4% to 15% [6,7]. In this population, these infections frequently involve the lower respiratory tract and may be associated with substantial morbidity and mortality [5]. Acute mortality in lower respiratory tract infection has been reported in up to 6–20% of cases, whereas overall mortality has been estimated at 10–20% despite medical treatment and supportive care [8].

Importantly, RVIs have also been implicated in long-term graft outcomes. Several studies have suggested that RVI, particularly those involving the lower respiratory tract, may increase the risk of chronic lung allograft dysfunction (CLAD), a major determinant of long-term survival after LT [1]. RSV may be particularly associated with higher risk of CLAD [9]. The proposed mechanisms include virus-induced epithelial injury, immune activation, and subsequent alloimmune responses, although the precise pathogenic pathways remain incompletely understood.

Despite the clinical impact of RSV and hMPV infections, their optimal management remains uncertain. In contrast to influenza, for which antiviral treatment strategies are well established, therapeutic approaches for RSV and hMPV in transplant recipients are heterogeneous and often center-specific [5]. Treatment of RSV may include oral or aerosolized ribavirin, immunomodulators (immunoglobulins or antibody-based treatment such as palivizumab) or steroids, with limited evidence regarding their efficacy.

Ribavirin is a broad-spectrum nucleoside analogue with activity against RNA viruses including RSV, hMPV and Parainfluenza virus, and has been widely used in this setting [5]. However, evidence supporting its clinical benefit remains limited and is largely derived from observational studies, which hampers a robust assessment of its effectiveness [5]. Aerosolized ribavirin is cumbersome and expensive to administer, limiting its routine use in clinical practice. Oral ribavirin, although easier to administer, has been associated with potential toxicities, including hemolytic anemia, leukopenia, and neuropsychiatric symptoms [3]. Despite this, real-world studies suggest that both oral and inhaled ribavirin are generally well tolerated. Oral ribavirin is therefore considered a practical alternative to the aerosolized formulation [10,11].

Management is heterogeneous across centers, reflecting reliance on single-center retrospective studies. The clinical impact of ribavirin remains uncertain, underscoring the need for multicenter real-world data. This study aimed to describe the clinical presentation, management patterns, and longitudinal outcomes of RSV and hMPV infections in lung transplant recipients, with particular attention to real-world use of ribavirin-containing treatment strategies and their association with post-infection outcomes.

## 2. Materials and Methods

### 2.1. Study Design

We conducted a multicenter prospective, observational cohort study, enrolling LT patients between September 2021 and March 2024 across 5 LT centers in Spain (12 de Octubre University Hospital, La Fe de Valencia University Hospital, Puerta de Hierro University Hospital, Valdecilla University Hospital, Vall d’Hebron University Hospital). Participants were followed for up to 1 year or until earlier termination due to dropout or death. Written informed consent was obtained from all participants, or when necessary, from a legally authorized representative. The study protocol was approved by the institutional review boards at each participating center and was evaluated by the Spanish Agency of Medicines and Medical Devices (AEMPS).

### 2.2. Participants

Adult lung transplant recipients aged ≥18 years were eligible if RSV or hMPV was detected in an upper or lower respiratory tract sample using a polymerase chain reaction (PCR)-based nucleic acid amplification assay. Only microbiologically confirmed episodes were included. Exclusion criteria were age < 18 years, absence of microbiological confirmation, and inability to obtain informed consent from the patient or a legally authorized representative. Pregnant women were excluded, and women of childbearing potential were required to have a negative pregnancy test before inclusion.

Respiratory symptoms were identified during scheduled or unscheduled clinical assessments or were reported by patients to the lung transplant team. Patients with respiratory symptoms, fever of unknown origin, or pneumonia during epidemic seasonal RVI underwent nasopharyngeal swab testing. Bronchoalveolar lavage (BAL) was performed when upper respiratory samples were negative or when lower respiratory tract involvement was suspected, and during protocol bronchoscopies.

Respiratory viral testing was performed when clinically indicated according to local practice rather than systematic screening. Microbiological diagnosis was established using nucleic acid amplification assays that included both RSV and hMPV, performed on upper or lower respiratory tract samples according to the clinical presentation. In cases of co-detection of multiple viruses, all identified viruses were recorded.

RVI episodes were classified at the time of diagnosis as asymptomatic, upper respiratory tract infection, or lower respiratory tract infection according to ECIL-4 guidelines [12]. Lower respiratory tract disease was defined “as pathological sputum production, hypoxia, or pulmonary infiltrates together with identification of community-acquired respiratory virus in respiratory secretions” [12]. Final classification was based on the treating physician’s assessment after consideration of alternative causes and concomitant infections.

Management during the acute phase followed local standard-of-care practices without protocol-driven intervention. Clinical management was initiated at diagnosis according to local practice. Importantly, ribavirin was not administered according to a predefined protocol but was prescribed at the discretion of the treating physician, in accordance with institutional practice, mainly in patients considered at higher risk of complications or suspected lower respiratory tract involvement within the first 7 days after diagnosis. When prescribed, treatment consisted of an initial intravenous dose of 30 mg/kg on day 1, followed by oral ribavirin at 30 mg/kg/day divided into three doses. Adjunctive therapies, including corticosteroids, antibiotics, or intravenous immunoglobulins, were prescribed at the discretion of the treating physician. Patients not receiving ribavirin were managed with supportive care and/or adjunctive treatments according to clinical indication.

In patients presenting with a decline in lung function and no alternative identifiable cause, further diagnostic evaluation was performed at the discretion of the treating physician. When clinically indicated, multiple transbronchial biopsies were obtained to assess for acute cellular rejection. Acute cellular rejection was defined according to the International Society for Heart and Lung Transplantation (ISHLT) classification when histological confirmation was available [13]. Anti-rejection treatment could also be administered in selected cases with very high clinical suspicion after exclusion of other potential causes of deterioration in lung function, according to the treating physician’s judgment [13].

### 2.3. Data Collection

Clinical, treatment, and outcome data were prospectively collected from electronic medical records at diagnosis, at day 14, at 90 days and at 1 year after infection. Data on clinical presentation, physical examination findings, laboratory results, infection management (including treatment modalities, dosing, and ribavirin use and related adverse events as specified in the summary of product characteristics), were systematically recorded, along with longitudinal clinical and lung function outcomes.

### 2.4. Outcomes

The primary outcome was the association between ribavirin use and the percentage change in forced expiratory volume in the first second (FEV_1_) at 90 days after infection, calculated relative to the FEV_1_ value measured 3 months before infection [14]. Secondary outcomes included acute lung function decline, defined as a ≥10% decrease in FEV_1_ at 14 days after infection, FEV_1_ change at 1-year, acute cellular rejection, CLAD onset or progression, recurrent respiratory infections, hospital admission, oxygen requirement, ICU admission, and mortality.

### 2.5. Ethics

The study was approved by the institutional review boards or ethics committees of all participating centers, and was conducted in accordance with the Declaration of Helsinki. Written informed consent was obtained from all participants.

### 2.6. Statistical Analysis

Descriptive statistics were used to summarize the baseline characteristics of the study participants. Categorical variables are presented as numbers and percentages. Continuous variables are reported as mean ± standard deviation (SD) or median and interquartile range (IQR), depending on the distribution assessed using the Shapiro–Wilk test. Comparisons between groups were performed using the chi-square test or Fisher’s exact test for categorical variables, and Student’s *t*-test or Mann–Whitney U test for continuous variables, as appropriate. Changes in FEV_1_ before and during infection were evaluated using Wilcoxon signed-rank tests.

A multivariable linear regression model was used to evaluate the association between ribavirin treatment and 90-day percentage FEV_1_ change from the pre-infection baseline. The model included ribavirin treatment, lower respiratory tract infection, baseline FEV_1_ measured three months before infection, pre-existing CLAD [15], and time since lung transplantation. Exploratory interaction analyses were performed between ribavirin treatment and each covariate. The ribavirin-by-baseline FEV_1_ interaction was the only interaction showing statistical evidence of heterogeneity and was retained in the final model. The final model retained the main effects for ribavirin treatment, lower respiratory tract infection, baseline FEV_1_, pre-existing CLAD, and time since lung transplantation, together with the ribavirin-by-baseline FEV_1_ interaction. Because of this interaction, the effect of ribavirin was interpreted using estimated marginal effects across clinically relevant baseline FEV_1_ values, as shown in Supplemental Material Figure S1. Propensity score methods were considered but not performed because treatment allocation showed insufficient overlap across centers. Accordingly, the estimates were interpreted as adjusted associations rather than causal treatment effects.

Missing data were not imputed. Descriptive analyses were based on available observations, and the multivariable regression was performed as a complete-case analysis.

A two-sided *p* value < 0.05 was considered statistically significant. Statistical analyses were performed using Stata version 15 (StataCorp, College Station, TX, USA).

### 2.7. Artificial Intelligence

Artificial intelligence tools were used during the preparation of this manuscript for grammar correction and language refinement. All content was subsequently reviewed and validated by the authors to ensure accuracy and scientific integrity.

## 3. Results

**Figure 1:**
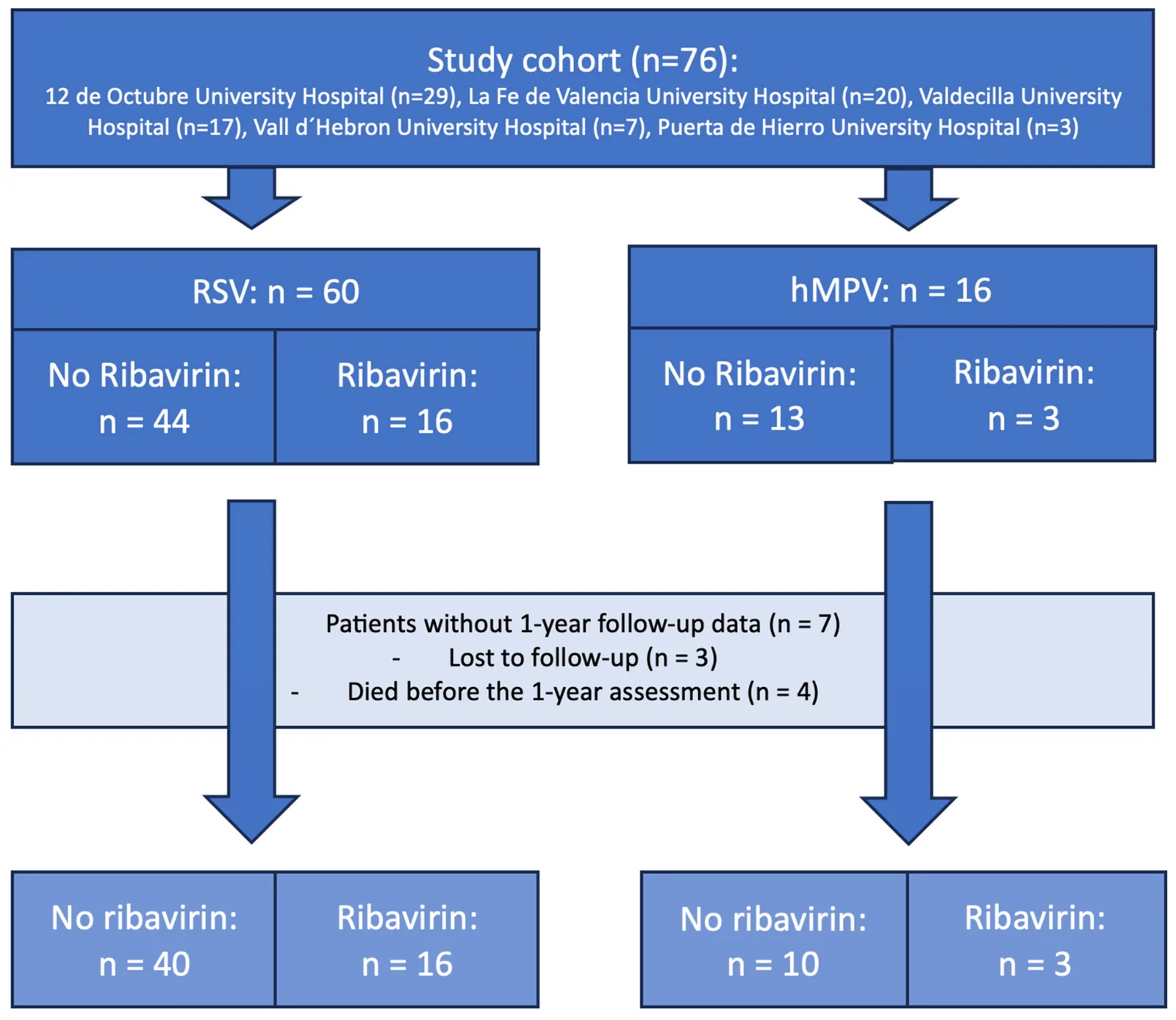
Flow diagram of the study cohort, treatment groups, and availability of 1-year follow-up data. hMPV: human metapneumovirus. RSV: respiratory syncytial virus.

A total of 76 LT recipients with RSV or hMPV infection were included in the study. The flow of patients through the study and treatment groups is shown in Figure 1.

### 3.1. Patient Characteristics

The cohort included 76 patients, of whom 40 were women (52.6%) with a mean age of 56.3 years (SD 14.0). The mean time since LT was 3.3 years (SD 2.7). Most patients had undergone bilateral LT (94.7%; *n* = 72). The most common underlying disease was interstitial lung disease (ILD) (36 patients, 47.4%) followed by chronic obstructive pulmonary disease (27 patients, 35.3%). Most patients (*n* = 64, 84.2%) were receiving a triple immunosuppressive regimen consisting of calcineurin inhibitors (tacrolimus *n* = 68, 93.2%; cyclosporine *n* = 5, 6.9%), an antimetabolite (mycophenolate *n* = 52, 71.2%), mTOR inhibitor (everolimus *n* = 13, 17.8%) and prednisone (*n* = 73). At the time of diagnosis, 27.6% of patients had CLAD distributed as follows: grade 0 (*n* = 4), grade 1 (*n* = 8), grade 2 (*n* = 3), and grade 3 (*n* = 6).

### 3.2. Infection Characteristics

A total of 60 patients were infected with RSV and 16 with hMPV. Their clinical characteristics, management, and outcomes according to respiratory virus are summarized in Table 1. Infections occurred a mean of 3.0 years after LT (SD 2.7). Diagnosis was established using a nasopharyngeal swab in 61 patients (82%) and BAL in 13 (17.6%). Lower respiratory tract symptoms were present in 37 patients (48.7%) and 4 patients (5.3%) were asymptomatic. Pneumonia occurred in 6 patients (8.3%). The median time from symptoms onset to diagnosis was 7 days (IQR 5–8). Overall, 40 patients (52.6%) required hospital admission, with a median length of stay of 11 days (IQR 6–18). Among hospitalized patients, supplemental oxygen was required in 23 (56.1%). Of these, 22 received low-flow oxygen therapy with a fraction of inspired oxygen (FiO_2_) below 36%, while one required extracorporeal membrane oxygenation. Two patients were admitted to the intensive care unit admission.

During the acute phase, empirical antibiotics were administered in 65 patients (87.8%), systemic corticosteroids in 44 (61.1%), and intravenous immunoglobulins in 14 (19.4%). Among corticosteroid-treated patients, regimens varied according to center and individual clinical assessment. Most patients received a prednisone-equivalent dose of 0.5 mg/kg/day, four received 1 mg/kg/day, and five received a 500-mg intravenous methylprednisolone bolus.

**Figure A1:**
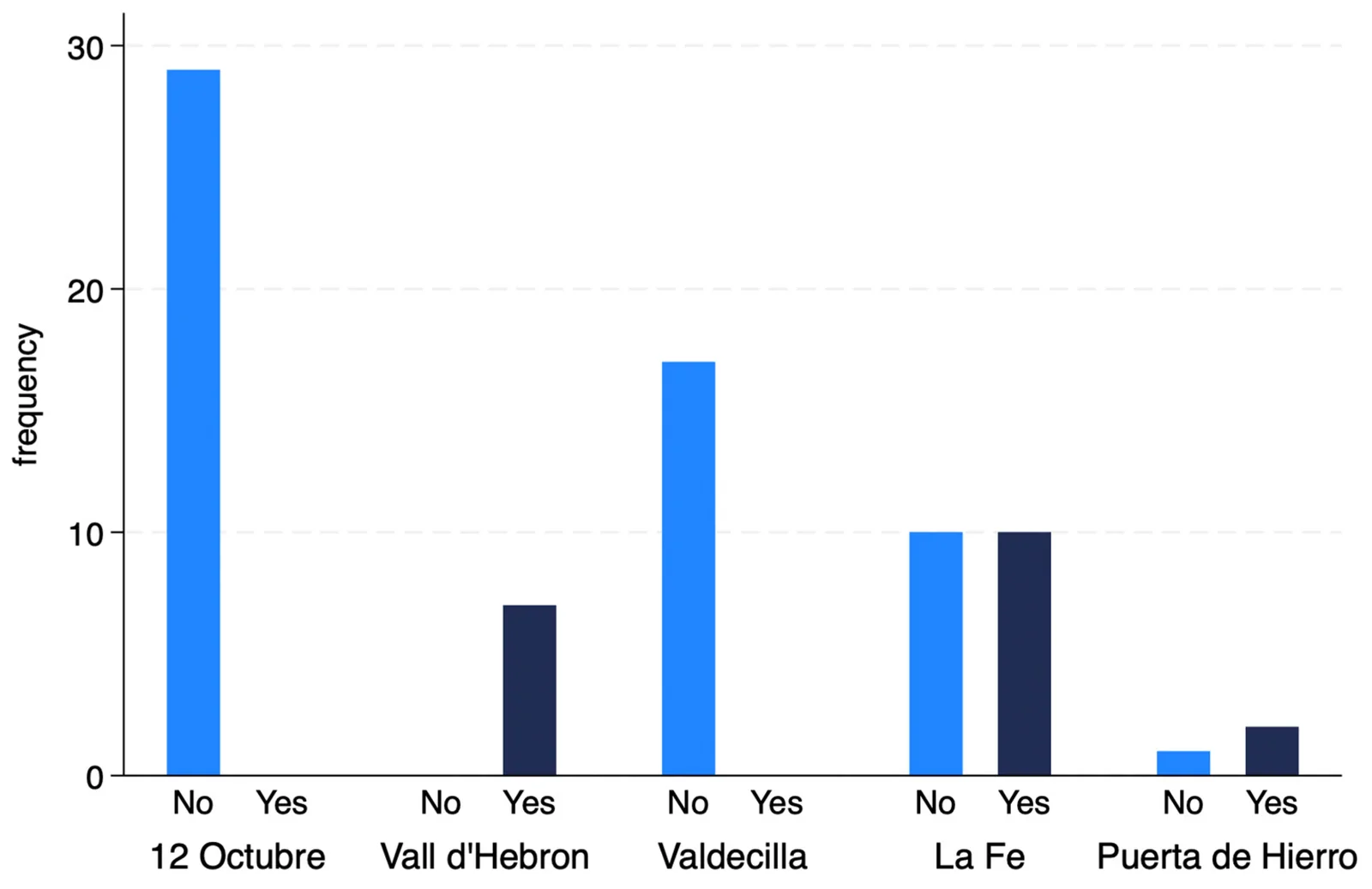
Patterns of ribavirin use across different centers in patients with RSV or hMPV infection. RSV: Respiratory syncytial virus. hMPV human metapneumovirus.

Ribavirin was prescribed in 19 patients overall (25%) across 3 centers (Figure A1), including 16 patients with RSV infection (26.7%) and 3 with hMPV infection (18.8%). All patients receiving ribavirin were concomitantly treated with systemic corticosteroids (100% vs. 47.1% in the non-ribavirin group; *p* < 0.001). The median duration of ribavirin therapy was 7 days (IQR 5–10). Two patients developed hemolytic anemia as an adverse effect. Among the 37 patients diagnosed with lower respiratory tract infection, 14 (37.8%) received ribavirin and steroids, whereas 17 (45.9%) were treated with corticosteroids alone.

Baseline characteristics of patients who received ribavirin compared with those who did not are shown in Table 2.

### 3.3. Evolution

FEV_1_ measurements were available in 65 patients at the pre-infection baseline, 65 at day 14, 67 at 90 days, and 67 at 1 year. The percentage change in FEV_1_ at 90 days could be calculated in 58 patients, who constituted the complete-case sample for the multivariable analysis.

At day 14 post-infection, median FEV_1_ change from baseline (3 months pre-infection) was −55 mL (IQR −210 to 70), with 17 patients (29.3%) showing a >10% decline. Acute cellular rejection occurred in 3 patients (4.2%), and 11 patients developed co-infections (7 viral, 2 bacterial, and 2 fungal). No deaths were observed during this period. Table 3 summarizes clinical outcomes across treatment groups (ribavirin plus steroids, steroids alone, or no treatment), while Table 4 presents the characteristics and follow-up of patients with lower respiratory tract infection stratified by treatment (steroids alone vs. steroids plus ribavirin).

**Figure 2:**
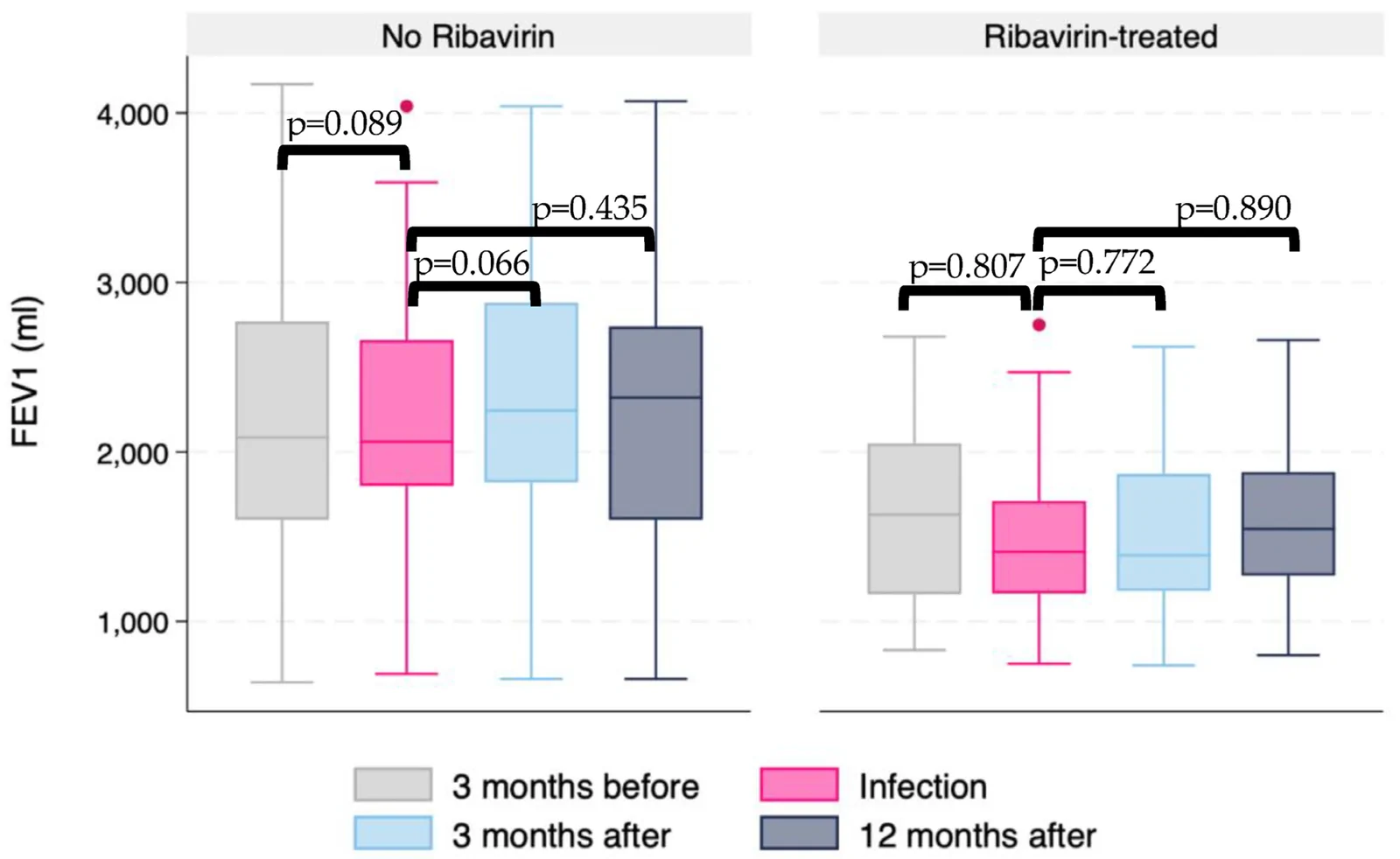
Evolution of lung function before and after infection over 1-year follow-up, stratified by ribavirin treatment. FEV_1_: forced expiratory volume in the first second.

**Figure A2:**
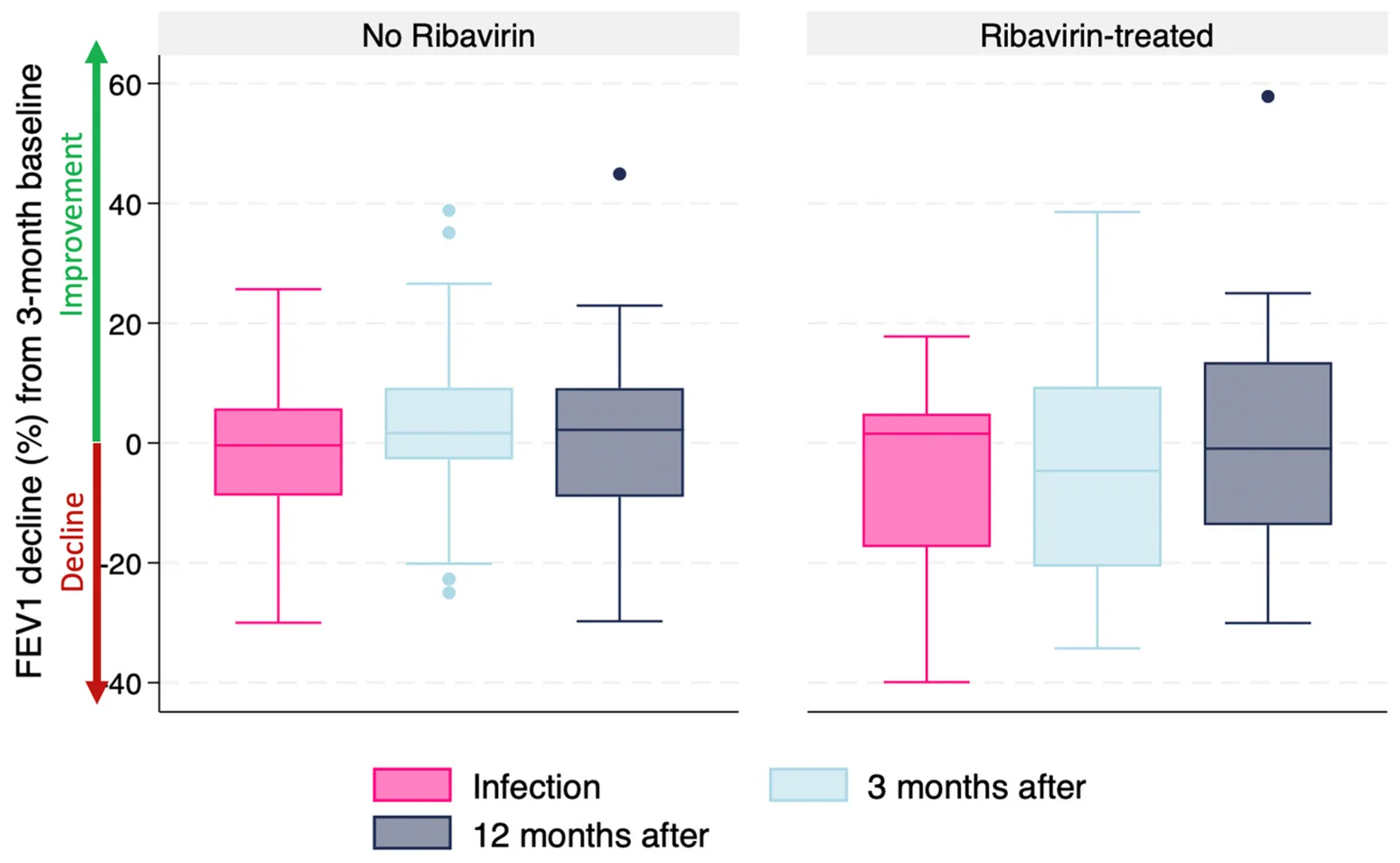
Percentage change in Forced expiratory volume in the first second (FEV_1_) relative to the value measured 3 months before infection.

Lung function during follow-up is summarized in Figure 2 and Figure A2.

In multivariable linear regression analysis evaluating factors associated with the percentage decline in FEV_1_ at 90 days (Table 5), ribavirin treatment was not independently associated with the outcome when interpreted at the reference value of baseline FEV_1_ (β = 27.49; 95% CI, −2.99 to 57.97; *p* = 0.076). However, a significant interaction between ribavirin treatment and baseline FEV_1_ was observed (β for interaction = −0.0183, *p* = 0.027), indicating that the estimated association with ribavirin became less favorable as baseline FEV_1_ increased. Marginal effects analysis showed more favorable estimates at lower baseline FEV_1_ values. Pre-existing CLAD showed a trend toward a greater decline in FEV_1_ (β = −10.57, *p* = 0.06), whereas baseline FEV_1_ and time since transplantation were not independently associated with the outcome. Overall, the explanatory performance of the model was modest, with an adjusted R^2^ of 0.16.

During the first-year follow-up, five patients developed CLAD (4 in the ribavirin-treated group) and 12 experienced progressions of pre-existing CLAD (8 in the ribavirin group). Four patients developed acute cellular rejection, all of whom had received ribavirin. Four patients died during follow-up, with deaths attributed to causes unrelated to the initial infection.

At 3-month follow-up, nine patients were diagnosed with tracheobronchitis, and four had microbiological isolation (two viral, one bacterial and one fungal). After one year of follow-up, three patients developed pneumonia (4.5%), 11 tracheobronchitis (16.7%), and nine had other microbiological isolations (4 viral, 3 bacterial, 1 fungal, and 1 viral–fungal co-infection).

## 4. Discussion

This multicenter real-world study shows heterogeneity in the management of RSV and hMPV infections after LT. In this observational cohort, ribavirin use was not independently associated with improved lung function outcomes; however, the study was not designed to establish treatment efficacy or causality. In our cohort, ribavirin was administered in approximately one-quarter of cases, and its use varied across participating centers, with ribavirin routinely prescribed at three centers.

Ribavirin was more frequently administered to patients requiring hospitalization, receiving adjunctive therapies such as corticosteroids and immunoglobulins, and undergoing bronchoalveolar lavage for diagnosis, indicating its preferential use in patients with more severe clinical presentations. Notably, all patients treated with ribavirin also received systemic corticosteroids, compared with approximately half of untreated patients, suggesting that ribavirin was incorporated into a more intensive therapeutic approach rather than used as a standalone intervention. In addition, acute cellular rejection within 14 days was more frequent among ribavirin-treated patients. This finding should be interpreted cautiously, as it likely reflects the higher-risk profile of patients selected for treatment rather than a direct effect of ribavirin. Exploratory analyses accounting for corticosteroid use did not show meaningful differences in lung function outcomes across treatment groups (Table 3), supporting that corticosteroid co-administration is unlikely to fully explain the observed results.

Although ribavirin has shown promising results in small observational studies, larger analyses have reported conflicting findings regarding its clinical benefit. Our findings are consistent with this uncertainty. Despite the in vitro susceptibility of RSV and hMPV to ribavirin, this antiviral activity may not translate into consistent clinical benefit across different clinical scenarios [5]. In the adjusted analysis, ribavirin use was not independently associated with the percentage change in FEV_1_ at 90 days after infection. This finding should not be interpreted as evidence of lack of efficacy, because treatment allocation was non-random, the sample size was limited, and patients receiving ribavirin had a more severe clinical profile. The ribavirin-by-baseline FEV_1_ interaction suggested a more favorable adjusted association at lower pre-infection FEV_1_ values. However, no established biological mechanism explains this finding, which may also reflect the influence of baseline FEV_1_ on percentage change, regression to the mean, residual confounding, or chance. Given the small sample size and exploratory nature of the analysis, this observation should be considered hypothesis-generating rather than evidence of benefit in patients with lower baseline FEV_1_.

Although a substantial proportion of patients experienced an acute decline in FEV_1_ after infection, no sustained deterioration in lung function was observed during follow-up, consistent with prior studies [7,10,11]. Similarly, ribavirin use was not associated with a lower incidence of CLAD during follow-up, in line with prior evidence [5]. Baseline prevalence of CLAD was similar between groups, suggesting that subsequent outcomes were not driven by pre-existing graft dysfunction. Although CLAD progression appeared more frequent in the ribavirin group, this likely reflects confounding by indication, given its preferential use in more severe cases, and contrasts with previous reports by Gottlieb [10] and Glanville [11]. To account for this, multivariable analysis adjusted for baseline lung function, pre-existing CLAD, and time since transplantation was performed.

We also observed low mortality during the acute phase of infection, which is in keeping with previous literature reporting relatively low short-term mortality associated with RSV and hMPV infections in LT recipients [5]. At the same time, these infections were still associated with relevant morbidity, including frequent hospital admission, oxygen requirement, and acute declines in lung function, underscoring their clinical relevance in this population.

In our cohort, hMPV infections were more frequently diagnosed using bronchoalveolar lavage samples. However, respiratory sampling was clinically driven, and paired upper and lower respiratory tract samples were not systematically obtained. Therefore, this finding may reflect selection for bronchoscopy rather than differences in the diagnostic yield of upper respiratory tract testing and should be interpreted cautiously. More generally, lower respiratory tract sampling may be considered in selected lung transplant recipients when upper respiratory tract testing is negative or lower respiratory tract involvement is suspected [16].

Prevention remains a key component of management in this population. In addition to infection control measures, the recent development of RSV vaccines may contribute to reducing the burden of these infections in transplant recipients, although their role in LT populations requires further evaluation [2].

This study has several limitations. Most importantly, its observational design and non-random treatment allocation substantially limit causal inference regarding ribavirin. Ribavirin was preferentially administered to patients with more severe or clinically concerning presentations, including lower respiratory tract involvement, hospitalization, bronchoalveolar lavage-based diagnosis, and receipt of intravenous immunoglobulin. Moreover, all ribavirin-treated patients also received systemic corticosteroids, making it difficult to disentangle the independent association of ribavirin from disease severity and concomitant treatment. Although multivariable adjustment was performed, residual and unmeasured confounding by indication cannot be excluded [5]. Propensity score methods were explored but were not considered reliable because treatment allocation showed inadequate overlap across centers. The limited overall sample size, particularly the small number of ribavirin-treated patients, also reduced statistical power and the precision of effect estimates for outcomes such as CLAD, rejection, and mortality. Therefore, the absence of a statistically significant association should not be interpreted as evidence that ribavirin is ineffective. Finally, pooling RSV and hMPV infections may have obscured virus-specific differences in clinical presentation and outcomes. Although exploratory comparisons were performed, the small number of hMPV cases precluded a reliable assessment of virus-specific treatment associations or a treatment-by-virus interaction.

Other limitations should also be acknowledged. Classification of lower respiratory tract infection included an element of clinical judgment and may therefore have been partly subjective. Although this was a multicenter study including centers with broadly similar maintenance immunosuppression and infection-management protocols, differences in ribavirin use and recruitment across centers may reflect local practices and seasonal viral circulation. Parainfluenza virus infections were not included in the prespecified study population; therefore, the findings cannot be extrapolated to lung transplant recipients with parainfluenza infection. The study was conducted before the implementation of current RSV vaccination recommendations in solid organ transplant recipients, and its findings may not fully reflect outcomes in vaccinated populations. Finally, ribavirin concentrations were not measured; therefore, insufficient pulmonary exposure during oral treatment cannot be excluded. Limited pharmacokinetic evidence suggests lower intracellular ribavirin triphosphate concentrations in bronchoalveolar lavage cells with oral than with inhaled administration, although therapeutic target concentrations in the respiratory compartment remain undefined [17].

Despite these limitations, this study represents one of the largest multicenter real-world cohorts of LT recipients with RSV or hMPV infection and provides relevant information on current management and outcomes in routine practice. Our findings support the idea that acute-phase treatment remains non-standardized and that the benefit of ribavirin remains uncertain in this setting. Further multicenter studies with larger sample sizes and more homogeneous definitions and management strategies are needed to better define the role of antiviral treatment in these infections.

## 5. Conclusions

In conclusion, RSV and hMPV infections in LT recipients were associated with substantial morbidity, particularly among patients with severe lower respiratory tract disease, but low infection-attributable mortality. Treatment approaches were heterogeneous, reflecting the lack of standardized management strategies in this setting. In this non-randomized real-world cohort, ribavirin use was not associated with improved lung function outcomes after adjustment, although residual confounding by indication remains likely. Larger prospective studies with standardized treatment strategies are needed to determine its potential clinical benefit.

## Figures and Tables

**Table 1:** Clinical characteristics, management, and outcomes according to respiratory virus.

		RSV *n* = 60 (78.9%)	hMPV *n* = 16 (21.1%)	*p* Value
Baseline characteristics	Age (years), mean (SD)	56.8 (12.7)	54.8 (18.4)	0.614
	Time since LT (years), median (IQR)	3.3 (0.8–4.8)	2.9 (1.4–6.4)	0.563
	Women, *n* (%)	35 (58.3)	5 (31.2)	0.05
	Bilateral LT, *n* (%)	58 (96.7)	14 (87.5)	0.186
	Pre-existing CLAD, *n* (%)	16 (26.7)	5 (31.3)	0.758
	FEV_1_ 3 months before infection (mL), median (IQR)	2060 (1470–2650)	1720 (1220–1990)	0.100
	FEV_1_ 3 months before infection (% of peak LT FEV_1_), median (IQR)	87.2 (78.1–98)	83.0 (60.7–87.8)	0.069
Clinical presentation	Asymptomatic presentation, *n* (%) Lower respiratory tract infection, *n*(%) Pneumonia, *n* (%)	2 (3.4) 30 (50.9) 4 (6.9)	2 (12.5) 7 (43.8) 2 (12.5)	0.191 0.614 0.399
	Diagnostic sample, *n* (%) - Nasopharyngeal swab - Bronchoalveolar lavage	47 (81.0) 11 (19.0)	14 (87.5) 2 (12.5)	0.534
	SpO_2_ (%) at diagnosis, median (IQR)	97 (92–98)	94.5 (92.5–96)	0.287
Management	Intravenous immunoglobulin, *n* (%)	8 (14.3)	6 (37.5)	0.050
	Steroids, *n* (%)	34 (67.1)	10 (62.5)	0.897
	Antibiotics, *n* (%)	50 (86.2)	15 (93.8)	0.383
	Ribavirin, *n* (%)	16 (26.7)	3 (18.8)	0.506
	Admission, *n* (%)	30 (54.4)	10 (62.5)	0.561
	Admission (days), median (IQR)	9 (6–21)	11 (6–15)	0.860
Outcomes	Percent change in FEV_1_ from pre-infection baseline, median (IQR) - at 90-day follow-up - at 1-year follow-up	−0.3 (−11.3–7.6) −0.1 (−14.2–9.2)	0.1 (−7.0–12.7) 1.3 (−3.6–6.2)	0.590
	Acute rejection, *n* (%) - at 14-day follow-up - at 90-day follow-up - at 1-year follow-up	2 (3.5) 1 (1.8) 0	1 (7.1) 0 0	0.570 0.516
	CLAD onset, *n* (%) - at 90-day follow-up - at 1-year follow-up	2 (3.6) 4 (7.4)	0 1 (7.7)	0.357 0.972
	CLAD progression, *n* (%) - at 90-day follow-up - at 1-year follow-up	4 (7.4) 5 (9.3)	2 (15.4) 1 (7.7)	0.375 0.857
	Status at 1-year follow-up - Death, *n* (%) - Lost to follow-up, *n* (%)	1 (1.7) 3 (5.0)	3 (18.8) 0	0.031

CLAD: chronic lung allograft dysfunction. FEV_1_: forced expiratory volume in first second. mL: milliliters. IQR: interquartile range. LT: lung transplant. SD: standard deviation. SpO_2_: peripheral oxygen saturation.

**Table 2:** Baseline characteristics of patients according to ribavirin treatment.

	Ribavirin *n* = 19 (25%)	No Ribavirin *n* = 57 (75%)	*p* Value
Age (years-old), mean (SD)	58.9 (12.3)	55.4 (14.5)	0.353
Years after lung transplant, median (IQR)	1.7 (0.7–4.5)	3.5 (1.0–5.1)	0.276
Women, *n* (%)	9 (47.4)	31 (54.4)	0.596
Underlying disease, *n* (%) - Interstitial lung diseases - COPD - Cystic fibrosis/bronchiectasis - Pulmonary hypertension	8 (42.1) 8 (42.1) 3 (15.8) 0	28 (49.1) 19 (33.3) 6 (10.5) 4 (7.0)	0.382
Bilateral lung transplant, *n* (%)	18 (94.7)	54 (94.7)	1.000
Triple immunosuppressive therapy, *n* (%)	17 (89.5)	47 (84.0)	0.778
Pre-existing CLAD, *n* (%) - Grade 0 - Grade 1 - Grade 2 - Grade 3	2 (10.5) 2 (10.5) 2 (10.5) 2 (10.5)	2 (3.5) 6 (10.5) 1 (1.8) 4 (7.0)	0.351
Respiratory virus, *n* (%) - Respiratory syncytial virus - Metapneumovirus	16 (84.2) 3 (15.8)	44 (77.2) 13 (22.8)	0.506
Diagnostic sample, *n* (%) - Nasopharyngeal swab - Bronchoalveolar lavage	11 (57.9) 8 (42.1)	50 (90.9) 5 (9.1)	0.002
FVC 3 months before infection (mL), median (IQR)	2565 (2300–2800)	2920 (2350–3510)	0.211
FEV_1_ 3 months before infection (mL), median (IQR)	1695 (1250–2130)	1990 (1470–2650)	0.142
FEV_1_ 3 months before infection (% of maximum post LT), median (IQR)	75.5 (61.5–95)	87.1 (78.1–95)	0.245
Symptoms, *n* (%) - Asymptomatic - Lower respiratory tract infection	2 (10.5) 14 (73.7)	2 (3.6) 23 (41.1)	0.264 0.014
SpO_2_ (%) at diagnosis, median (IQR)	94 (92–98)	96 (92.5–98)	0.358
Leukocytes (cells/µL), median (IQR)	7850 (5640–10,090)	6500 (4400–7900)	0.148
Intravenous immunoglobulin treatment, n (%)	8 (42.1)	6 (11.3)	0.006
Steroids, *n* (%)	19 (100)	25 (47.1)	<0.001
Antibiotics, *n* (%)	17 (89.4)	48 (87.3)	0.798
Admission, *n* (%)	18 (94.7)	22 (42.6)	<0.001
Length of hospital stay (days), median (IQR)	14.5 (7–27)	7 (3–14)	0.045

CLAD: chronic lung allograft dysfunction. COPD: chronic obstructive pulmonary disease FEV_1_: forced expiratory volume in first second. FVC: forced vital capacity. mL: milliliters. IQR: interquartile range. LT: lung transplant. SD: standard deviation. SpO_2_ peripheral oxygen saturation.

**Table 3:** Clinical outcomes in RSV/hMPV-infected patients by treatment strategy (ribavirin plus steroids, steroids alone, or no treatment), among patients with available treatment-strategy data (n = 72).

	Ribavirin + Steroids *n* = 19 (25%)	Steroids Only *n* = 25 (34.5%)	No Ribavirin or Steroids *n* = 28 (38.9%)	*p* Value
Percent change in FEV_1_ from pre-infection baseline, median (IQR) - at 90-day follow-up - at 1-year follow-up	−4.6 (−20.6–9.4) −0.9 (−4.8–20)	−0.4 (−8.0–10.6) −0.6 (−9.0–5.5)	0.7 (−6.7–7.9) 0.8 (−14.2–9.6)	0.707 0.106
Respiratory infections, *n* (%) - at 90-day follow-up - at 1-year follow-up	1 (5.3) 2 (8.0)	1 (4.0) 4 (16.0)	2 (7.1) 3 (10.8)	1.000 0.829
Pneumonia, *n* (%) - at 90-day follow-up - at 1-year follow-up	0 3 (15.8)	0 0	0 0	0.018
Acute rejection, *n* (%) - at 14-day follow-up - at 90-day follow-up - at 1-year follow-up	3 (15.8) 1 (5.3) 0	0 0 0	0 0 0	0.016 0.262 —
CLAD onset, *n* (%) - at 90-day follow-up - at 1-year follow-up	0 1 (5.3)	2 (8.0) 1 (4.0)	0 2 (7.1)	0.176 1.000
CLAD progression, *n* (%) - at 90-day follow-up - at 1-year follow-up	4 (21.1) 4 (21.1)	0 0	2 (7.1) 1 (3.6)	0.037 0.029
Death, *n* (%) - at 1-year follow-up	0	2	2	0.775

CLAD: chronic lung allograft dysfunction. FEV_1_: forced expiratory volume in first second. CLAD onset was evaluated only in patients without baseline CLAD, while CLAD progression was assessed among patients with pre-existing CLAD.

**Table 4:** Characteristics and outcomes among patients with lower respiratory tract infection who received systemic corticoisteroids (*n* = 31) according to ribavirin use treatment (ribavirin plus steroids (*n* = 14) vs. steroids alone (*n* = 17)).

	Ribavirin + Steroids *n* = 14	Steroids *n* = 17	*p* Value
Age (years-old), median (IQR)	61.4 (50.8–65.7)	61.7 (56.5–64.8)	0.812
Years after lung transplant, median (IQR)	1.4 (0.7–3.5)	3.9 (0.9–6.1)	0.110
Women, *n* (%)	6 (42.5)	8 (47.1)	0.551
Bilateral lung transplant, *n* (%)	13 (92.9)	17 (100)	0.452
Triple immunosuppressive therapy, *n* (%)	12 (85.7)	14 (82.4)	0.597
Pre-existing CLAD grade, *n* (%): - Grade 0 - Grade 1 - Grade 2 - Grade 3	1 (7.1) 1 (7.1) 1 (7.1) 2 (14.3)	1 (5.9) 1 (5.9) 2 (11.8) 0	0.941
Respiratory virus, *n* (%) - Respiratory syncytial virus - Metapneumovirus	12 (85.7) 2 (14.3)	13 (76.5) 4 (23.5)	0.664
FEV_1_ (% of peak post–LT FEV_1_) measured 3 months prior, median (IQR)	68.5 (61.5–79.5)	85.9 (78–94)	0.07
SpO_2_ (%) at diagnosis, median (IQR)	94.5 (92.0–98.0)	95.0 (91.0–98.0)	0.759
Leukocytes (cells/µL), median (IQR)	7660 (5640–11,840)	6390 (4500–8050)	0.308
Immunoglobulin treatment, *n* (%)	6 (42.9)	6 (35.3)	0.475
Antibiotics, *n* (%)	13 (92.9)	17 (100)	0.452
Admission, *n* (%)	13 (92.9)	11 (64.7)	0.073
Admission (days), median (IQR)	14 (7–21)	8 (3–14)	0.129
Drop of FEV_1_ > 10% from 3 months prior at 14 days after infection, *n* (%)	1 (11.1)	6 (42.9)	0.124
Acute rejection, *n* (%) - at 14-day - at 1-year follow up	1 (7.7) 0	0 0	0.464
CLAD onset, *n* (%) - at 90-day - at 1 year follow-up	0 1 (7.7)	1 (6.7) 1 (7.7)	0.566 0.760
CLAD progression, *n* (%) - at 90-day - at 1-year follow-up	1 (8.3) 3 (23.1)	0 0	0.444 0.110
One-year follow-up status, *n* (%): - death - lost	0 0	2 (11.8) 1 (5.9)	0.145

CLAD: chronic lung allograft dysfunction. FEV_1_: forced expiratory volume in first second. IQR: interquartile range. SpO_2_ peripheral oxygen saturation.

**Table 5:** Multivariable linear regression analysis of factors associated with the percentage change in FEV_1_ decline at 90 days after infection.

	Beta Coefficient (95% CI)	*p* Value
Ribavirin treatment (yes/no)	27.50 (−2.99 to 57.98)	0.076
Lower respiratory tract infection (yes/no)	2.04 (−6.08–10.17)	0.608
FEV_1_ 3 months before infection (mL), median (IQR)	−0.001 (−0.006 to 0.005)	0.860
Pre-existing CLAD (yes/no)	−10.57 (−21.62 to 0.47)	0.060
Time since transplantation (years)	−1.36 (−3.15 to 0.44)	0.135
Ribavirin x baseline FEV_1_	−0.02 (−0.03 to −0.00)	0.027

Model characteristics: N = 58; R^2^ = 0.25; adjusted R^2^ = 0.17; F (6,51) = 2.78; *p* = 0.021. The effect of ribavirin should be interpreted considering the interaction with baseline FEV_1_. CLAD: chronic lung allograft dysfunction. FEV_1_: forced expiratory volume in first second.

## Data Availability

The data supporting the findings of this study are not publicly available due to privacy and ethical restrictions.

## Supplementary Materials

The following supporting information can be downloaded at: https://www.mdpi.com/article/10.3390/life16081323/s1, Figure S1: Adjusted marginal association of ribavirin therapy with the 90-day percentage change in FEV_1_ relative to baseline FEV_1_. Adjusted marginal association between ribavirin use and the percentage change in FEV_1_ at 90 days across baseline FEV_1_ values. Points represent the estimated adjusted difference between ribavirin-treated and untreated patients, and vertical bars represent 95% confidence intervals. Positive values indicate a more favorable estimated FEV_1_ change among ribavirin-treated patients, whereas the horizontal line at zero indicates no estimated difference between groups. These findings are exploratory and should not be interpreted as evidence of treatment benefit within specific baseline FEV_1_ subgroups. FEV_1_: forced expiratory volume in the first second.

## Abbreviations

The following abbreviations are used in this manuscript:

AEMPS	Spanish Agency of Medicines and Medical Devices
BAL	Bronchoalveolar lavage
CI	Confidence interval
CLAD	Chronic lung allograft dysfunction
COPD	Chronic obstructive pulmonary disease
ECIL-4	Fourth European Conference on Infections in Leukemia
FEV_1_	Forced expiratory volume in the first second
FiO_2_	Fraction of inspired oxygen
FVC	Forced vital capacity
hMPV	Human metapneumovirus
ICU	Intensive care unit
ILD	Interstitial lung disease
IQR	Interquartile range
LT	Lung transplant
mTOR	Mechanistic target of rapamycin
RNA	Ribonucleic acid
RSV	Respiratory syncytial virus
RVI	Respiratory viral infection
SD	Standard deviation
SpO2	Peripheral oxygen saturation
