# Supplementary material for: Longitudinal Outcomes and Ribavirin Use in Lung Transplant Recipients with Respiratory Syncytial Virus or Human Metapneumovirus Infection: A Real-World Multicenter Cohort Study
Source: Life (Basel). 2026 Aug 12;16(8):1323. doi: 10.3390/life16081323 (PMC13514294; doi:10.3390/life16081323)
Supplement: Supplementary file 1 [file life-16-01323-s001.zip › life-4426823-supplementary.pdf]

Supplemental Material

Figure S1. Adjusted marginal association of ribavirin therapy with the 90-day percentage change in FEV1 relative to baseline FEV1.

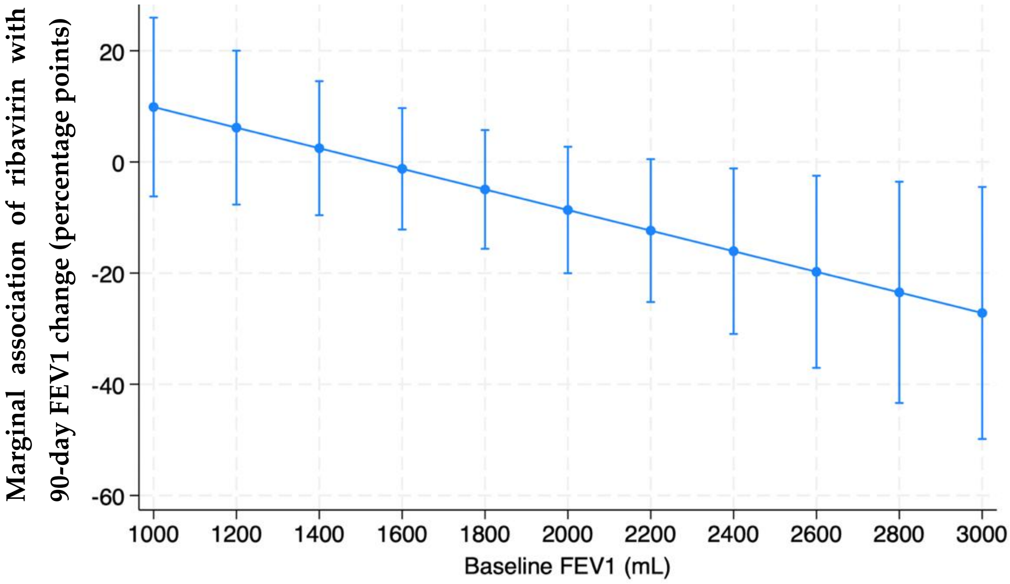

Adjusted marginal association between ribavirin use and the percentage change in FEV_1 at 90 days across baseline FEV_1 values. Points represent the estimated adjusted difference between ribavirin-treated and untreated patients, and vertical bars represent 95% confidence intervals. Positive values indicate a more favorable estimated FEV_1 change among ribavirin-treated patients, whereas the horizontal line at zero indicates no estimated difference between groups. These findings are exploratory and should not be interpreted as evidence of treatment benefit within specific baseline FEV_1 subgroups. FEV_1: forced expiratory volume in the first second.

https://doi.org/10.3390/life16081323
